# Preliminary research on the effect of sutra chanting on oral and respiratory function: a comparison between expert sutra chanting buddhist priests and general buddhist priests in Japan

**DOI:** 10.3389/fragi.2025.1632997

**Published:** 2025-09-26

**Authors:** Ayako Edahiro, Chiaki Ura, Yoshiko Motohashi, Ryosho Shoji, Reisai Kaneko, Yukan Ogawa, Akinori Takase, Kousho Nakano, Tsuyoshi Okamura

**Affiliations:** 1 Tokyo Metropolitan Institute for Geriatrics and Gerontology, Tokyo, Japan; 2 Jodo Shu Research Institute, Tokyo, Japan; 3 Shingon Shu Buzan-ha, Chiba, Japan; 4 Institute of Regional Development, Taisho University, Tokyo, Japan; 5 Faculty of Socio-Symbiosis, Taisho University, Tokyo, Japan

**Keywords:** oral function, respiratory function, hyoid excursion, sutra chant, buddhism

## Abstract

**Introduction:**

There is limited prior research on the physiological effects of sutra chanting.

**Methods:**

The health effects of sutra chanting were explored by comparing the oral and respiratory functions of Buddhist priests who are experts in sutra chanting with those of general Buddhist priests. In addition to basic characteristics, lifestyle variables, and general health status, participants underwent assessment of oral function and respiratory function by two certified dentists.

**Results:**

Compared to general priests (n = 23), expert chanters (n = 49) were significantly higher in peak expiratory flow (PEF), forced vital capacity (FVC), and hyoid displacement (⊿HD). In the two multiple regression analyses which include PEF and FVC as the dependent variables, expert group demonstrated significantly better function.

**Discussion:**

Considering its historical and cultural background, the idea of using sutra chanting has potential in a healthcare program for older people at risk of declining oral and respiratory functions.

## 1 Introduction

Mindfulness is an accepted intervention in older adults with various health concerns (Hazlett-Stevens et al., 2019) and Japanese Buddhism is the origin of mindfulness (Kabat-Zinn, 2003). Zazen, which has influenced mindfulness, is one of the two most important practices in Japanese Buddhism. Sutra chanting is another important practice of Japanese Buddhism.

**FIGURE 1:**
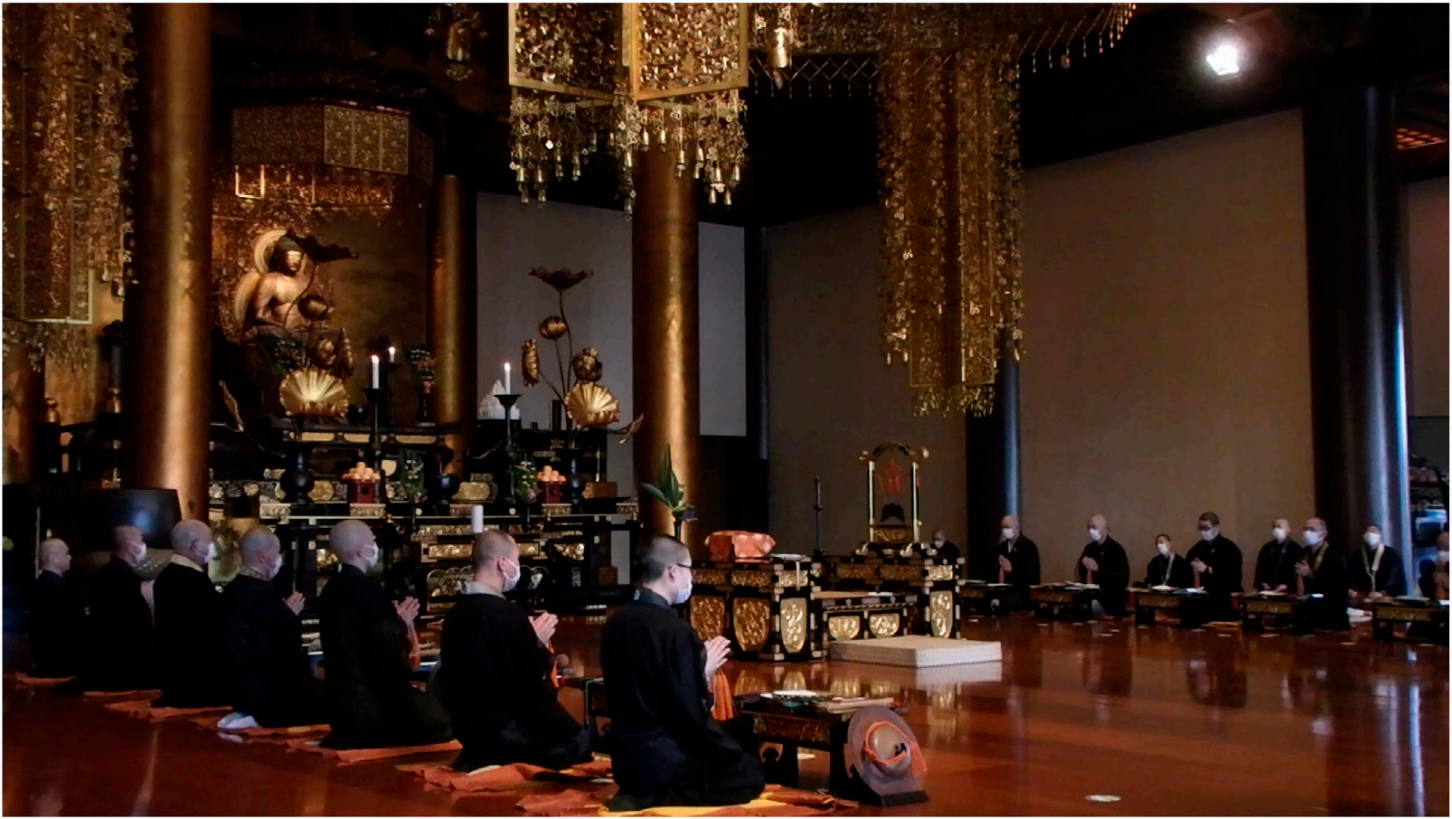
Religious ceremony in which experienced priests chant a Sabdavidya “shomyo” offering to the Buddha.

In Japan, Buddhist practitioners are encouraged to perform repetitive and loud recitations of Buddhist sutras, which contain teachings, every morning in search of mental peace. Listening to sutra chanting reduces bereavement stress (Taniyama et al., 2021). Sabdavidya *shomyo* (声明) (Seroussi, 2002) is the chanting of Buddhist hymns by experienced priests, the traditional sound of voices used in religious ceremonies as offerings to Buddha (Figure 1). Chanting involves a special vocalization method that uses an extended breathing technique with a fixed rhythm and pitch. According to oral science, sutra chanting may have health benefits. However, compared to Zen meditation, research on the effect of sutra chanting on health outcomes is limited.

Generally, respiratory function decreases with aging (Thomas et al., 2019) and the incidence of aspiration pneumonia is high among older adults in Japan (Teramoto et al., 2008). The Pa-Ta-Ka-Ra exercise is commonly used to prevent aspiration pneumonia (Hara et al., 2012). In this exercise, tongue movements are used to generate sounds as repeated syllables (e.g., Ta-ta-ta-ta-ta and Ka-ka-ka-ka-ka) that are characteristic of Japanese linguistic constructions. In addition, older adults at risk of oral hypofunction and dysphagia are asked to perform repetitive exercises involving syntactic sound sequences that are not part of daily words (e.g., Pa-ta-ka-ra). However, clinically, patients often state that meaningless vocalizations are boring. Vocalization has favorable effects. Moreover, singing is used in respiratory rehabilitation (Kim et al., 2023). Karaoke, a popular interactive leisure activity worldwide following the development of the karaoke machine in Japan in the 1970s, involves long phonation and reportedly im-proves respiratory function (Miyazaki and Mori, 2020).

In 2010, the number of Buddhists worldwide was 488 million, representing 7% of the world’s population (Pew Research Center, 2012). Considering its popularity among Buddhists, if sutra chanting is related to better oral and respiratory function, it could expand the possibility of developing a novel intervention for older adults, especially in Asia.

As there is limited prior research on the physiological effects of *shomyo* sutra chanting, this exploratory study was designed to investigate the effects of sutra chanting by comparing the oral and respiratory functions of expert Buddhist priests with those of general Buddhist priests.

## 2 Materials and methods

### 2.1 Participants

The participants comprised Buddhist priests who were expert (expert group) or nonexpert (control group) in *shomyo* sutra chanting. *Shomyo* is a special vocalization method using very long breathing techniques with a fixed rhythm and pitch, which is the chanting of Buddhist hymns for religious ceremonies. Among the priests of the Jodo denomination, a group of priests called Shiki-shi perform these religious musical ceremonies. The experts in this study are not defined by specific objective criteria; rather, they are the Shiki-shi priests who specialize in *shomyo* sutra chanting. They practice diligently every day, gather twice a year for joint practice sessions, and participate in an annual presentation event to refine their ceremonial chanting skills throughout the year. The expert priests who participated in our study were those who came to Tokyo for one of these biannual practice sessions at Zojoji Temple, the head temple of the Jodo denomination in eastern Japan. The control group was recruited from Zojoji Temple, where they worked as managers of the denomination.

The expert group consisted solely of male priests, which was determined by historical factors and beyond our control. Therefore, we did not recruit female priests as controls.

The expert group was measured on 29 June 2022 and the control group on 3 October 2022. One month before the measurement, potential participants were informed of the date and location of the survey and asked to come to the venue.

### 2.2 Measures

#### 2.2.1 Oral function

Objective assessment of oral health status was performed by two dentists as described below; Participants underwent an oral examination that included the number of teeth and denture use, conducted by two dentists.

General oral health status was assessed using the Oral Health Assessment Tool - Japanese Version (OHAT-J) (Matsuo and Nakagawa, 2016). OHAT-J is an evaluation tool that assesses the lips, tongue, gums and tissues, saliva, natural teeth, dentures, oral cleanliness, and dental pain through comprehensive observational evaluation (Chalmers et al., 2005), and is widely used internationally.

Oral diadochokinesis (ODK) was assessed by instructing participants to rapidly and repeatedly articulate the monosyllables/pa/,/ta/, and/ka/for 5 s. The number of articulations was recorded and converted into a score expressed as repetitions per second (Sakayori et al., 2013). A digital counter (T.K.K. 3,350; Takei Rika Kikai, Niigata, Japan) was used to perform the ODK test, which serves as an index of lip and tongue motor function.

Occlusal pressure was measured using Dental PrescaleⅡ^®^ (GC, Tokyo, Japan), which is a pressure-sensitive sheet to measure occlusal pressure (Hara et al., 2020). The sheet contains microcapsules that rupture under pressure, releasing dye to visualize contact points between the upper and lower teeth in varying shades of red—darker red indicating stronger contact. The sheets were scanned using an image scanning system (Bite Force Analyzer, GC, Tokyo, Japan) to quantify both the total occlusal pressure and the pressure exerted in the denture or natural tooth areas. Measurements were taken under the following standardized conditions: the participant wore dentures, sat in an upright position, held the dorsum of the tongue parallel to the floor, and bit down with maximal force for 3 s.

Oral moisture was measured using the oral moisture-checking device (Mucus^®^, Life Co., Saitama, Japan), which evaluates electrostatic capacity based on impedance caused by high-frequency waves (Takahashi et al., 2006). This method reflects both surface and subsurface (up to 50 μm deep) mucosal moisture. Measurements were taken at the center of the tongue’s underside, 10 mm from the tip, using a disposable-covered sensor applied with approximately 200 g of pressure. Three measurements were taken, and the median value was used (Fukushima et al., 2019).

The maximum phonation time (MPT) was used to evaluate exhalation and vocal function because it has been found to have a significant relationship with swallowing and coughing functions (Zhou et al., 2012). A cutoff value of 10 s is commonly used. The measurement was performed by having the subject sit upright and sustain the sound “ah” for as long as possible, with the duration recorded. The pitch was at a natural speaking level, and the intensity was at a natural moderate level, avoiding overly weak or strong voices. The measurement was conducted three times consecutively, and the maximum value was adopted.

**FIGURE 2:**
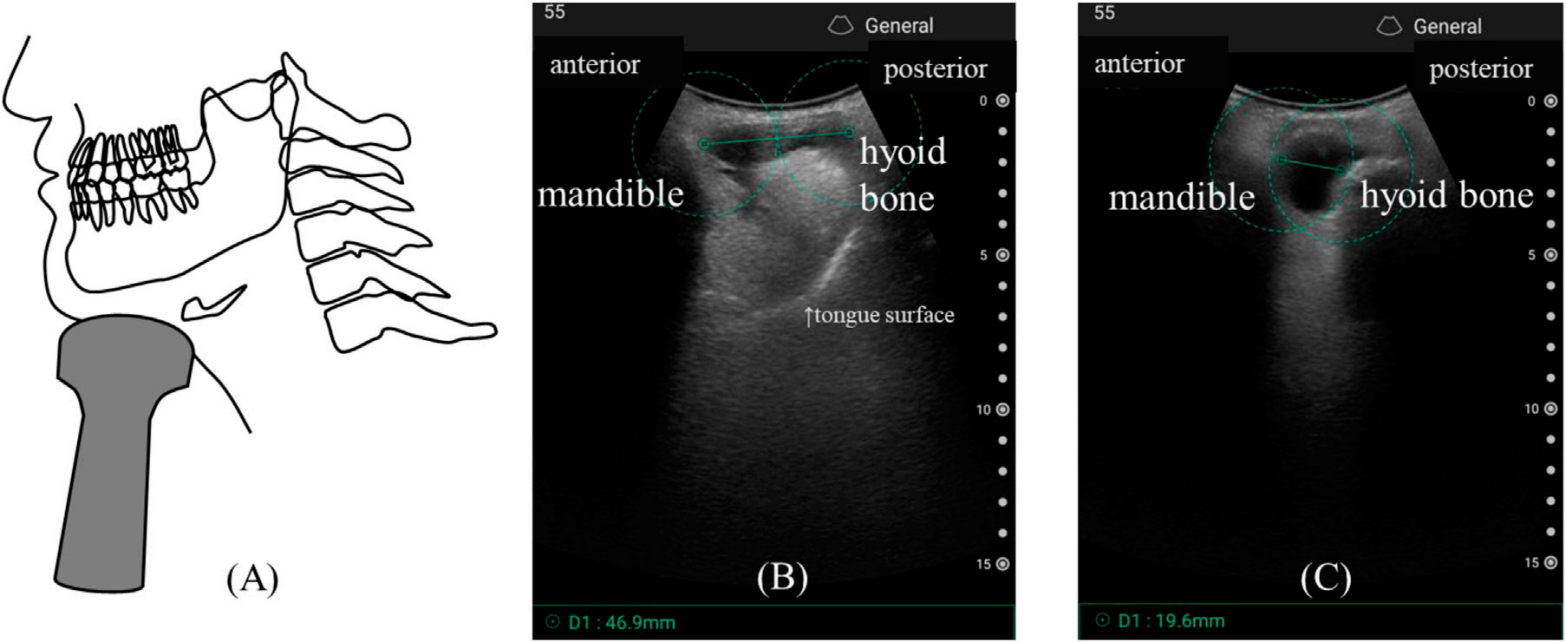
Ultrasound probe placement for assessing hyoid bone displacement **(A)**, and ultrasound images showing the hyoid bone’s resting position **(B)** and maximum displacement **(C)**. Measurements were performed using the caliper function in the FUJIFILM iViz air application. The straight-line distance from the mandibular border to the anterior border of the hyoid bone was measured according to Winiker’s technique. The upper shadow in the image represents the geniohyoid muscle.

Hyoid displacement (⊿HD) was measured using a portable ultrasound device (iViz Air convex type, Fujifilm, Japan) by a dentist certified in swallowing therapy. Participants were seated, facing forward, and instructed to perform a saliva effort swallow. The anatomical landmarks for measurement were the mandible, hyoid bone, and geniohyoid muscle. The probe was positioned in a sagittal direction, perpendicular to the geniohyoid muscle (Figure 2). For image acquisition, measurements were taken at two timings—at rest and during maximum displacement of a saliva effort swallow—for three sets, and a video was also recorded for confirmation. We used Winiker’s technique (Winiker et al., 2021). The ultrasound probe was positioned at a right angle to the mandible, geniohyoid muscle, and hyoid bone, ensuring that the hyoid bone was clearly visualized. Measurements were taken at a depth of 7–10 cm, using a frequency of 4 MHz and a single-focus setting, to determine the hyoid rest position and maximum displacement. ⊿HD (%) was calculated using the following formula for body size correction. The ⊿HD was calculated for each of the three measurement sets using the formula below, and the maximum ⊿HD value from these sets was used for analysis.

$$⊿\text{HD }\left(\%\right)=\text{hyoid displacement }\left(\text{HD}\right)\;/\text{ static distance}\times 100$$

Subjective oral health status was assessed using questions concerning having subjective oral difficulty in gargling, having subjective difficulty in chewing, and having subjective choking on swallowing fluids, with potential answers of yes and no.

#### 2.2.2 Respiratory function

We measured participants’ respiratory function using portable devices rather than full-specification spirometers, in order to avoid damaging the temple’s wooden furnishings. Forced vital capacity (FVC) was measured using a Portable Dry Spirometer (Tsutsumi KC, Japan). We asked participants to take a deep breath, place the mouthpiece in their mouth, exhale completely in one go, continue exhaling for at least 6 s, and do this 3 times. Peak expiratory flow (PEF) and forced expiratory volume in 1 s (FEV 1.0) were measured using an electrical peak-flow meter (Asuma One, Japan). We asked participants to take a deep breath, place the mouthpiece in their mouth, exhale completely in one go, and continue exhaling for at least 6 s. All three respiratory function tests were performed in a seated position with a device held parallel to the floor and with the nose clip. All of the three measurements were conducted by one dentist certified in swallowing function therapy. The maximum values were used for the analysis. The reliability of repeated measurements was adjusted in advance before the test was conducted.

#### 2.2.3 Other variables

We measured physical variables that may affect oral and respiratory functions, such as age, body weight (BW) (kg) and body height (BH) in meters, grip strength (kg), and smoking habits, using the Brinkman Index (Brinkman and Coates, 1963) and skeletal muscle mass index (SMI) as the indicator of frailty (Janssen et al., 2000). Grip strength was measured using a Smedley-type digital grip dynamometer (T.K.K.5401 GRIP-D; Takei Rika Kikai, Niigata, Japan) with the patient in a standing position. The dominant hand was determined as the preferred hand used to eat and write. Each measurement was taken twice on the dominant hand with an interval of at least 30 s between each measurement, and the maximum value was used for analysis. Skeletal muscle mass was measured using the body composition analyzer (InBody S10, Biospace Co., Ltd., Seoul, Korea). The SMI was calculated as the appendicular muscle mass in kilograms divided by the square of the height in meters. All participants’ skeletal muscle mass was measured while seated, at a fasting timing, with probes placed on both hands and feet.

In addition, respondents were asked about their weekly sutra chanting time using the options; “below 1 h”, “at least 1 h”, “at least 2 h”, “at least 4 h”, “at least 6 h”, “at least 8 h”, and “at least 10 h”. We adopted the minimum time spent on sutra chanting as a numerical value. Sutra-chanting time was binarized using a threshold of 6 h, We also asked their engagement in leisure activities involving their voices (e.g., karaoke or rock and roll band) other than sutra chanting.

Furthermore, we interviewed whether the participants had the following diseases; hypertension, cerebral stroke, heart disease, diabetes, hyperlipidemia, gastrointestinal disease, respiratory disease, kidney and prostate disorders, musculoskeletal disorders, cancer, immune disorder, depression, and Parkinson’s disease.

Finally, researchers confirmed that all subjects were not suffering from a cold and did not have a blocked nose.

**FIGURE 3:**
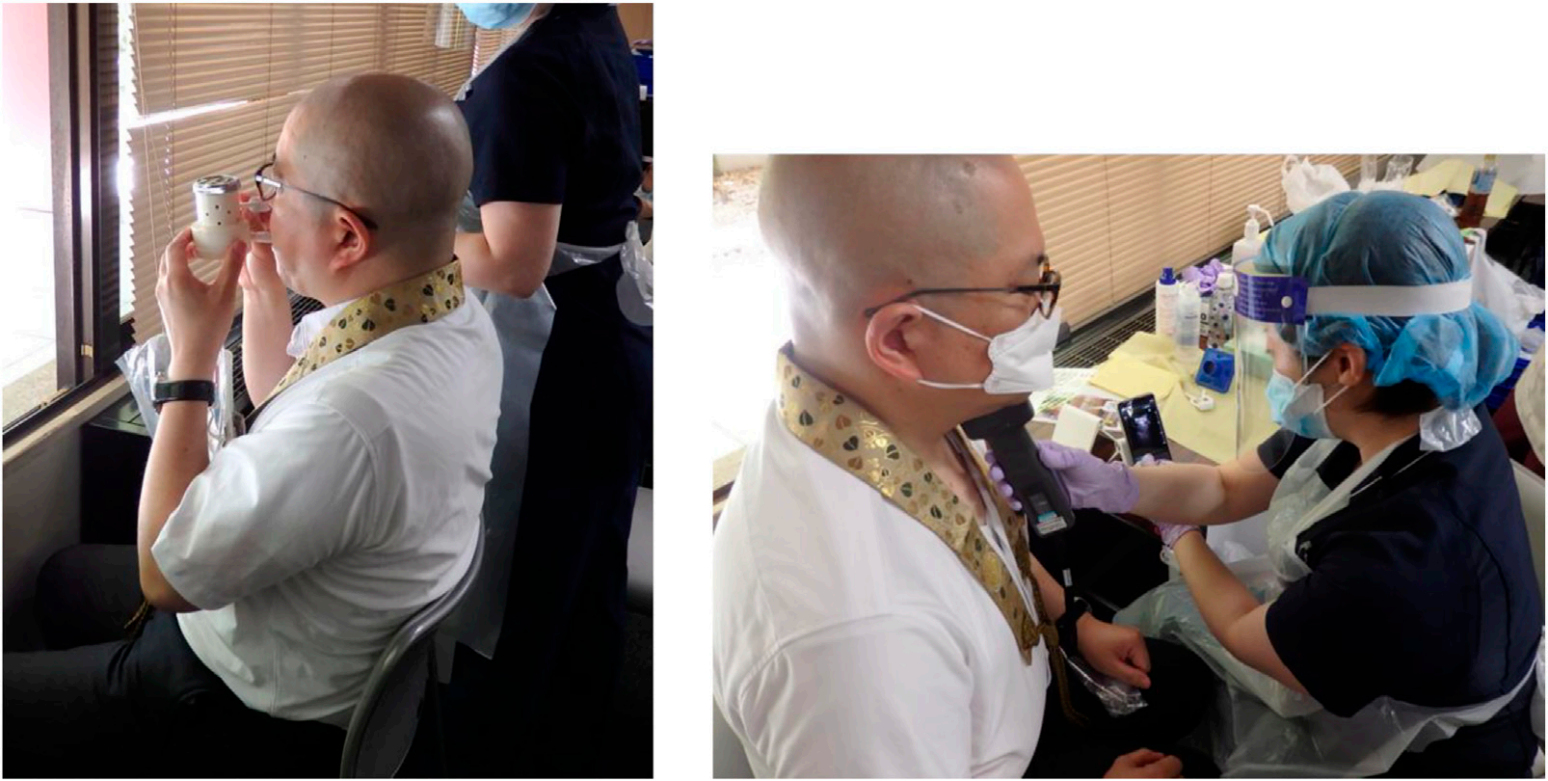
The dentist checked a Buddhist priest’s oral function, respiratory function (left), and hyoid bone displacement by ultrasonic examination (right).

The measurement setup is depicted in Figure 3.

### 2.3 Statistical analysis

In this study, MPT, ⊿HD, PEF, FEV1.0, and FVC were each measured three times, and the maximum value was used for the analysis. To ensure the validity of this approach, the consistency across the three measurements was examined using the intraclass correlation coefficient (ICC) and the standardized difference (Cohen’s d). Student’s t-test and the chi-squared test were used to analyze continuous variables and categorical data, respectively. Then, the items that showed statistical significance in the simple analysis from oral and respiratory function items were used as the dependent variable of multiple analyses.

A multiple regression analysis for PEF, FVC, and ⊿HD were conducted to confirm the effect of being in the expert group, adjusting for mediating variables. Because the number of participants did not allow for the inclusion of many variables in the multivariate analysis, we decided, after discussion with healthcare professionals, to include age, grip strength (kg), occlusal pressure (N), BH(m), smoking habit (Brinkman index), sutra-chanting time (>6 h), leisure activity using voice, and being in the expert priest group as independent variables. Variables generally related to respiratory and swallowing function, as well as those with potential influence within a priest’s activities, were selected and adopted after confirming for multicollinearity among the independent variables using the Variance Inflation Factor (VIF). Although SMI, which showed a significant difference between groups, was initially considered for inclusion in the model, a model including height was ultimately selected because it had a higher adjusted *R* ^2^. As a result, all independent variables had a VIF below 2.0, which is well below the generally acceptable threshold of 10, and therefore, multicollinearity was not considered a problem.

### 2.4 Positionality

The study team consisted of a dentist and an oral health researcher, a medical doctor, a psychologist, university professors who were Buddhist priests, and Buddhist priests from different denominations. None of the participants engaged in religious behavior or proselytizing. This study was not intended to benefit any particular religion or denomination; rather, to explore a brand-new method of promoting oral and respiratory wellbeing from existing resources. This study was funded by a secular research grant.

Furthermore, we do not intend to claim that religious practice is valuable due to medically verified evidence. Conversely, by evaluating medical research methodology and revealing the medical benefits of purely religious practices, we aimed to raise awareness in the medical community, which is faced with several challenges such as increasing patient numbers with respiratory pneumonia due to societal aging.

### 2.5 Ethical considerations

The research was completed in accordance with the Declaration of Helsinki as revised in 2013. The participants provided informed consent. The study protocol was approved by the Ethics Committee of Taisho University (approval number: 22–9).

Written informed consent was obtained from all individual participants included in the study. The authors affirm that human research participants provided informed consent for publication of the images included this manuscript, i.e., Figures 1, 3.

### 2.6 Data availability statement

The authors confirm that the data supporting the findings of this study are available within the article and its supplementary materials, which is available at 10.6084/m9. figshare.30112051.

## 3 Results

Seventy-two priests participated in this study, 49 and 23 in the expert and control groups, respectively. All participants were male, as explained above.

**FIGURE 4:**
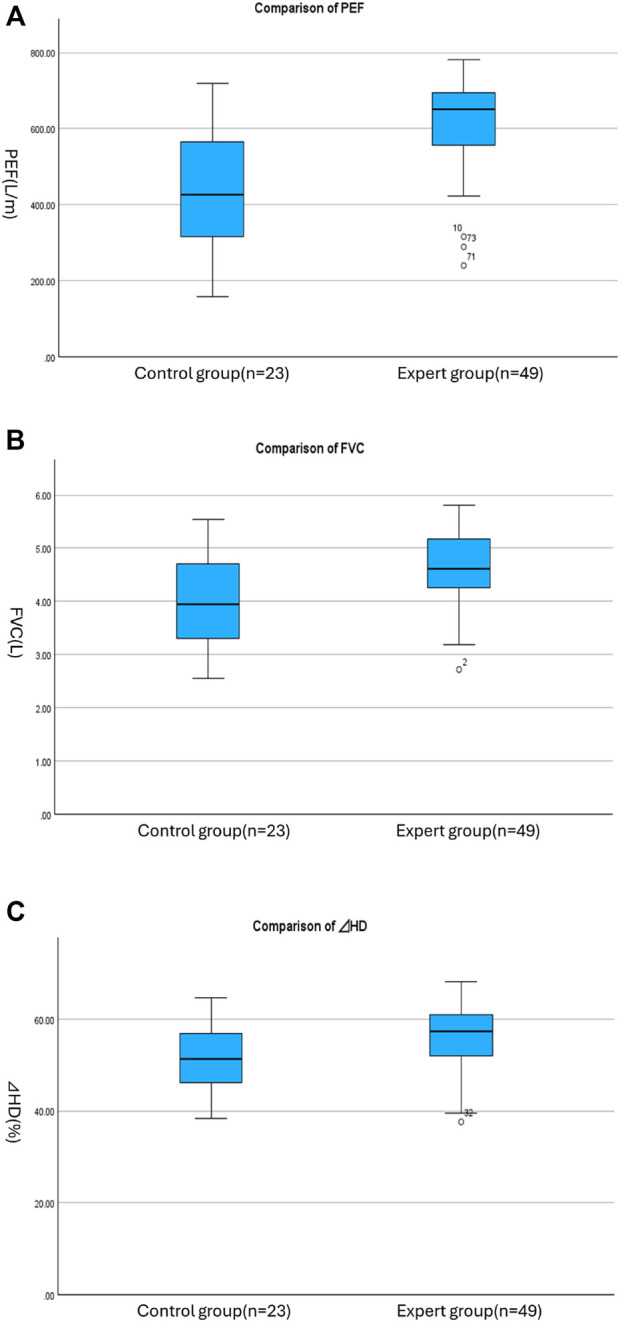
**(A–C)** Comparison of PEF, FVC, and ⊿HD between groups.

Concerning the oral functions, ⊿HD showed a significant difference (55.8 ± 6.9 vs51.4 ± 7.3, p = 0.015) between the two groups indicating that sutra chant experts have better swallowing function; however, number of teeth, denture use, OHAT, oral diadochokinesis, occlusal pressure, oral moisture, MPT, difficulty in gargling, difficulty in chewing, and choking on swallowing fluids did not show difference. Concerning the respiratory function, PEF (617.3 ± 125.4 vs. 443.8 ± 156.5, p < 0.001) and FVC (4.6 ± 0.7 vs. 4.0 ± 0.9, p = 0.001) showed a statistically significant difference (Figure 4). Basic characteristics such as age, BH, BW, BMI, grip strength, lifestyle variables that affect oral and respiratory function such as smoking, sutra-chanting time, having leisure activity using voice, and general health status did not differ between the two groups. Only the SMI showed the superiority of the expert group (8.7 ± 0.8 vs. 8.3 ± 0.6, p = 0.020). The comparative characteristics of the two groups are presented in Table 1.

**TABLE 1:** Comparative characteristics of Buddhist priests who are experts in *shomyo* sutra chanting (expert group) and Buddhist priests who are not (control group).

	Control group (n = 23)	Expert group (n = 49)	Total (n = 72)	p
Oral function				
Number of teeth (n)	28.2 ± 1.6	28.2 ± 2.5	28.2 ± 2.2	0.958
Denture use (%)	0.0%	4.1%	2.8%	0.460
OHAT-J	0.3 ± 0.6	0.5 ± 0.8	0.4 ± 0.7	0.445
ODK/pa/(n/s)	7.2 ± 0.8	7.3 ± 0.7	7.3 ± 0.7	0.418
ODK/ta/(n/s)	7.5 ± 0.8	7.6 ± 0.9	7.6 ± 0.9	0.881
ODK/ka/(n/s)	7.0 ± 1.0	7.0 ± 0.7	7.0 ± 0.8	0.968
Occlusal pressure (N)	1500.5 ± 612.2	1245.9 ± 520.1	1327.2 ± 559.7	0.072
Oral moisture	29.0 ± 2.4	27.9 ± 3.8	28.2 ± 3.4	0.185
MPT (s)	30.7 ± 6.8	34.1 ± 11.1	33.0 ± 10.0	0.181
⊿HD (%)	51.4 ± 7.3	55.8 ± 6.9	54.4 ± 7.3	0.015
Subjective difficulty in gargling (%)	0.0%	2.0%	1.4%	0.681
Subjective difficulty in chewing (%)	4.3%	4.1%	4.2%	0.691
Subjective choking on swallowing fluids (%)	17.4%	12.2%	13.9%	0.400
Respiratory Function				
PEF (L/min)	443.8 ± 156.5	617.3 ± 125.4	561.9 ± 157.6	<0.001
FEV1.0 (L)	3.3 ± 0.5	3.6 ± 0.5	3.5 ± 0.5	0.068
FVC (L)	4.0 ± 0.9	4.6 ± 0.7	4.4 ± 0.8	0.001
Basic Characteristics				
Age	36.4 ± 11.9	40.8 ± 10.2	39.4 ± 10.9	0.110
BH (m)	1.7 ± 0.1	1.7 ± 0.1	1.7 ± 0.1	0.851
BW (kg)	74.6 ± 10.6	79.0 ± 14.4	77.6 ± 13.4	0.194
BMI	25.0 ± 3.2	26.4 ± 4.4	25.9 ± 4.1	0.183
SMI (the appendicular muscle mass/BH*BH)	8.3 ± 0.6	8.7 ± 0.8	8.6 ± 0.8	0.020
Grip Strength (Kg)	41.0 ± 6.2	42.1 ± 6.2	41.7 ± 6.2	0.464
Life-style variables that affect oral and respiratory function				
Smoking (Brinkman Index)	108.6 ± 362.1	105.6 ± 169.7	106.6 ± 245.2	0.963
Sutra-chanting time (>6 h)	43.5%	36.7%	38.9%	0.384
Having leisure activity using voice (yes)	26.1%	22.4%	23.6%	0.476
General health status				
Hypertension	4.3%	10.2%	8.3%	0.370
Cerebral stroke	0.0%	0.0%	0.0%	—
Heart disease	0.0%	4.1%	2.8%	0.460
Diabetes	0.0%	0.0%	0.0%	—
Hyperlipidemia	0.0%	2.0%	1.4%	0.681
Gastrointestinal disease	0%	6.1%	4.2%	0.309
Respiratory disease	4.4%	4.1%	4.2%	0.691
Kidney and prostate disorders	0%	4.1%	2.8%	0.460
Musculoskeletal disorders	0%	0%	0%	—
Cancer	0%	0%	0%	—
Immune disorder	0%	2%	1.4%	0.681
Depression	8.7%	2%	4.2%	0.238
Parkinson’s disease	0%	0%	0%	—

OHAT-J, the Oral Health Assessment Tool - Japanese Version; ODK, diadochokinesis; MPT, maximum phonation time; ⊿HD, variation of hyoid displacement; PEF, peak expiratory flow; FEV, 1.0, forced expiratory volume in 1 s; FVC, forced vital capacity; BH, body height; BW, body weight; BMI, body mass index; SMI, skeletal muscle mass index.

p<0.05; **p < 0.01; ***p < 0.001.

Multiple regression analyses, including PEF, FVC, and ⊿HD as the dependent variables, were conducted to confirm the effect of the expert group, which was shown in simple analyses (Tables 2). In the two multiple regression analyses which include PEF and FVC as the dependent variables, expert group demonstrated significantly better function (p < 0.001 and p = 0.001). However, in the analysis which includes ⊿HD as the dependent variable, expert group showed only a nonsignificant trend (p = 0.052). Assessing the model fit for the multiple regression models for PEF, FVC, and ⊿HD, the coefficients of determination (*R* ^2^) were 0.414, 0.379, and 0.146, respectively. Furthermore, the adjusted R^2^values, which account for the number of variables, were 0.340, 0.300, and 0.038, respectively, demonstrating the validity of the model.

**TABLE 2:** Results of the analysis. Effect of expert group in 3 models of multiple regression analyses which includes PEF (Table 2a), FVC (Table 2b), and ⊿HD (Table 2c) as the dependent variables.

​	B	SE	β	t	p	Tolerance	VIF	​
a								
y-intercept	1026.955	458.817	​	2.238	0.029	​	​	​
Age	0.069	1.527	0.005	0.045	0.964	0.837	1.195	​
Grip strength (kg)	8.188	2.665	0.322	3.072	0.003	0.848	1.179	**
Occlusal pressure (N)	−0.028	0.028	−0.101	−1.015	0.314	0.944	1.060	​
BH (m)	−546.556	279.193	−0.206	−1.958	0.055	0.842	1.187	​
Smoking habit (brinkman index)	0.074	0.066	0.116	1.123	0.266	0.878	1.139	​
Being expert priest	162.394	34.056	0.484	4.768	<0.001	0.904	1.107	***
Sutra-chanting time (>6 h)	29.476	33.026	0.092	0.892	0.376	0.879	1.138	​
Having leisure activity using voice	58.438	36.547	0.159	1.599	0.115	0.946	1.057	​

​	B	SE	β	t	p	Tolerance	VIF	​
b								
y-intercept	−3.414	2.442	​	−1.398	0.167	​	​	​
Age	−0.011	0.008	−0.149	−1.372	0.175	0.837	1.195	​
Grip strength (kg)	0.037	0.014	0.281	2.606	0.011	0.848	1.179	*
Occlusal pressure (N)	0.000	0.000	−0.009	−0.090	0.929	0.944	1.060	​
BH (m)	3.564	1.486	0.259	2.399	0.019	0.842	1.187	*
Smoking habit (brinkman index)	0.000	0.000	−0.053	−0.497	0.621	0.878	1.139	​
Being expert priest	0.661	0.181	0.381	3.649	0.001	0.904	1.107	**
Sutra-chanting time (>6 h)	0.090	0.176	0.054	0.513	0.610	0.879	1.138	​
Having leisure activity using voice	0.111	0.194	0.058	0.573	0.569	0.946	1.057	​

​	B	SE	β	t	p	Tolerance	VIF
c							
y-intercept	42.684	25.536	​	1.672	0.100	​	​
Age	0.065	0.085	0.097	0.760	0.450	0.837	1.195
Grip strength (kg)	−0.132	0.148	−0.112	−0.888	0.378	0.848	1.179
Occlusal pressure (N)	−0.001	0.002	−0.104	−0.864	0.391	0.944	1.060
BH (m)	9.695	15.539	0.079	0.624	0.535	0.842	1.187
Smoking habit (brinkman index)	0.004	0.004	0.122	0.983	0.329	0.878	1.139
Being expert priest	3.757	1.895	0.243	1.982	0.052	0.904	1.107
Sutra-chanting time (>6 h)	−2.292	1.838	−0.155	−1.247	0.217	0.879	1.138
Having leisure activity using voice	−0.333	2.034	−0.020	−0.164	0.871	0.946	1.057

B, regression coefficients for non-standardized data; SE, standard error; β, regression coefficients for standardized data; t, t-value; p, p-value; VIF, variance inflation factor; SMI, skeletal muscle mass index; BH, body height.

*p < 0.05; **p < 0.01; ***p < 0.001.

To confirm the consistency of the three repeated measurements of MPT, ⊿HD, PEF, FEV1.0, and FVC, ICCs were calculated. The results showed high consistency across all measurement items. Specifically, the ICCs were as follows: MPT (ICC = 0.952, 95% CI [0.930–0.969]), ⊿HD (ICC = 0.771, 95% CI [0.771–0.850]), PEF (ICC = 0.948, 95% CI [0.923–0.966]), FEV1.0 (ICC = 0.902, 95% CI [0.855–0.936]), and FVC (ICC = 0.966, 95% CI [0.950–0.978]). Although potential learning effects and fatigue across the three functional tests were assumed, the standardized differences (Cohen’s d) were all small (<0.5; Cohen’s d = −0.29, −0.04, −0.19, −0.21, −0.37). Even when statistically significant differences were observed, their effects were judged not to be clinically meaningful. Based on this high consistency, the small effect sizes, and the international guideline recommendations (ATS/ERS) to adopt the maximum value from multiple measurements (Graham et al., 2019), we concluded that the use of maximum values in this study was appropriate.

## 4 Discussion

In this study, the oral and respiratory functions of Buddhist priests who are expert at chanting sutras and general priests were compared. The results of this study indicate that being a sutra chanting expert was related to having better oral and respiratory functions. To our knowledge, sutra chanting has rarely been assessed medically and scientifically, our results provided new insights into the relationship between health and religious customs despite its small sample size.

Engaging in activities that utilize oral and respiratory function has been reported to contribute to better health outcomes among older people. A fMRI study revealed increased activity in the right angular gyrus and the left lingual gyrus after the music therapy intervention (Satoh et al., 2015). Music therapy is regarded as a promising intervention method for people with Alzheimer’s disease in the psychosocial domain (Matziorinis and Koelsch, 2022). Similarly, reading aloud is reported to relate to better cognitive function (Kulason et al., 2018). Regarding studies that have used oral and respiratory function as an outcome measure, singing is used in respiratory rehabilitation (Kim et al., 2023). Karaoke, a popular interactive leisure activity worldwide following the development of the karaoke machine in Japan in the 1970s, involves long phonation and reportedly improves respiratory function (Miyazaki and Mori, 2020). However, to the best of our knowledge, there was no previous literature on the effect of traditional, cultural, and religious practices that use vocalization in the context of preventing aspiration pneumonia.

The reason Buddhism-based practices have been widely accepted as health-related techniques in cultures and regions outside of Buddhism stems from the characteristics of Buddhism. Zen Master Dogen (1200–1253), the founder of zazen in Japan, described zazen as a way to transcend: “To study the Buddha Way is to study the self. To study the self is to forget the self. To forget the self is to be verified by all things. To be verified by all things is to let the body and mind of the self and the body and mind of others drop off” (Dogen, 2024). Similarly, the Eiheiji Temple, the cathedral headquarters of the Soto denomination, which is the largest Zen denomination and established by Dogen in 1244, describes zazen as “the fundamental practice, and not for the purpose of obtaining a certification of spiritual enlightenment” (Eiheiji Temple, 2012). Zazen and its global variant, mindfulness, is widely accepted in other civilizations because, superficially, they are a methodology for forgetting the trivialities of the surroundings and addressing the self. The practice does not include the teachings of a transcendent other.

The situation is similar with regard to sutra-chanting. The present study is neutral with regard to Buddhism, as described in the positionality section; The Heart Sutra has been interpreted as having cognitive-behavioral elements that teaches the abandonment of adherence to preconceptions. Heart Sutra is not very religious as it rarely teaches the transcendent other. In fact, it is becoming popular in secularism, popular music, and spirituality with modern music technologies, including samplers, electric artists, and vocaloid software (Reehl, 2021). No official English translation of the Heart Sutra exists; however, a translation of the Vietnamese version by the popular Buddhist master, Thich Nhat Hanh (Thich Nhat Hanh, 2017), is available in English.

The findings of this study can be used for social implementation. Clinically, patients often state that meaningless vocalizations, such as the Pa-Ta-Ka-Ra exercise, are boring. However, a sutra chant has a significant meaning and its sequences involve sounds not used in daily communication. It is possible that healthcare programs involving sutra chanting and focusing on oral and respiratory rehabilitation—drawing on examples such as mindfulness meditation for mental illness, which has received international attention—may have potential as supplementary or alternative approaches to conventional healthcare programs.

For implementing sutra chanting in a healthcare program, it is necessary to develop a sutra chanting program which is acceptable for the broad range of people. Buddhism is often perceived as a philosophy and means of attaining mental peace, whereas religious neutrality is required for health programs. The Heart Sutra is a good candidate for developing a sutra chant program for older adults regardless of religion. It does not advocate the superiority of any particular religion and is very short, requiring about 5 min to complete.

This study is preliminary and exploratory, and potential confounding factors were not fully examined. In particular, we did not inquire about the daily activities of the priests in either group. On the other hand, the priests in both groups belong to their own home temples and likely lead similar lifestyles, with the exception that the expert group practices as needed, while the control group priests work at their denomination’s office during the day.

This study had some limitations. 1) A primary limitation is the small sample size, and future studies with larger sample sizes are needed. 2) The participants were Buddhist priests belonging to the temple headquarters of one of the seven major denominations of Japanese Buddhism. 3) Mental health variables that potentially affect the oral and respiratory function such as depression, anxiety, and subjective stress were not assessed. 4) Nutritional status was not assessed. 5) Respiratory function was assessed by one researcher. Because this study did not use spirometry and only conducted simplified measurements, a precise evaluation of FEV1.0/FVC and PEF/FVC was difficult. While no numerical abnormalities were found and no associations were observed with age, BMI, self-reported respiratory diseases, or smoking, we did not exclude subjects based on respiratory disease. A more detailed examination of this population would require a study using standard spirometry. On the other hand, although it has been reported that incense exposure increases airway hyperresponsiveness in mice (Yamamoto et al., 2021), the respiratory function of priests who are routinely exposed to incense has not been studied. This area warrants future investigation. 6) The experts were measured in June and controls in October, which might have affected the oral and respiratory function. 7) Assessor was not blinded, which suggests the risk of measurement bias. 8) Further studies are needed to investigate potential gender differences.

## 5 Conclusion

This preliminary and exploratory research suggests that Buddhist priests who are expert in sutra chanting have better oral and respiratory functions than general priests. To use sutra chanting as a healthcare program for older people at risk of declining oral and respiratory functions, there are several additional challenges to be overcome. First, it will be necessary to conduct a full-scale study involving broader measurements and blinding of the assessors. Second, because the cross-sectional design of this paper has inherent limitations in determining a causal relationship, interventional study with randomization or quasi-experimental testing is needed. Third, developing the program that uses sutra-chanting and that is acceptable for the broad range of people is essential.

## Data Availability

The datasets presented in this study can be found in online repositories. The names of the repository/repositories and accession number(s) can be found in the article/supplementary material.
