# Correction: Preliminary research on the effect of sutra chanting on oral and respiratory function: a comparison between expert sutra chanting buddhist priests and general buddhist priests in Japan

**DOI:** 10.3389/fragi.2025.1721074

**Published:** 2025-12-12

**Authors:** Ayako Edahiro, Chiaki Ura, Yoshiko Motohashi, Ryosho Shoji, Reisai Kaneko, Yukan Ogawa, Akinori Takase, Kousho Nakano, Tsuyoshi Okamura

**Affiliations:** 1 Tokyo Metropolitan Institute for Geriatrics and Gerontology, Tokyo, Japan; 2 Jodo Shu Research Institute, Tokyo, Japan; 3 Shingon Shu Buzan-Ha, Chiba, Japan; 4 Institute of Regional Development, Taisho University, Tokyo, Japan; 5 Faculty of Socio-Symbiosis, Taisho University, Tokyo, Japan

**Keywords:** oral function, respiratory function, hyoid excursion, sutra chant, buddhism

There was a mistake in Table 2 as published. The original content was divided into three separate tables, but during the editing process, they were merged into a single table, which has made the meaning more difficult to understand. Furthermore, currently, the asterisks (*, **, ***) indicating statistical significance are attached directly to the numerical values (e.g., in the VIF column). However, these markers are intended to apply to the entire row. Therefore, the asterisks should be placed outside the VIF column, in the right-hand margin of the table, rather than next to the numerical values. The corrected Table 2 appears below.

**TABLE 2:** Results of the analysis. Effect of expert group in 3 models of multiple regression analyses which includes PEF (Table 2a), FVC (Table 2b), and ⊿HD (Table 2c) as the dependent variables.

​	B	SE	β	t	p	Tolerance	VIF	​
a								
y-intercept	1026.955	458.817	​	2.238	0.029	​	​	​
Age	0.069	1.527	0.005	0.045	0.964	0.837	1.195	​
Grip strength (kg)	8.188	2.665	0.322	3.072	0.003	0.848	1.179	**
Occlusal pressure (N)	−0.028	0.028	−0.101	−1.015	0.314	0.944	1.060	​
BH (m)	−546.556	279.193	−0.206	−1.958	0.055	0.842	1.187	​
Smoking habit (brinkman index)	0.074	0.066	0.116	1.123	0.266	0.878	1.139	​
Being expert priest	162.394	34.056	0.484	4.768	<0.001	0.904	1.107	***
Sutra-chanting time (>6 h)	29.476	33.026	0.092	0.892	0.376	0.879	1.138	​
Having leisure activity using voice	58.438	36.547	0.159	1.599	0.115	0.946	1.057	​

​	B	SE	β	t	p	Tolerance	VIF	​
b								
y-intercept	−3.414	2.442	​	−1.398	0.167	​	​	​
Age	−0.011	0.008	−0.149	−1.372	0.175	0.837	1.195	​
Grip strength (kg)	0.037	0.014	0.281	2.606	0.011	0.848	1.179	*
Occlusal pressure (N)	0.000	0.000	−0.009	−0.090	0.929	0.944	1.060	​
BH (m)	3.564	1.486	0.259	2.399	0.019	0.842	1.187	*
Smoking habit (brinkman index)	0.000	0.000	−0.053	−0.497	0.621	0.878	1.139	​
Being expert priest	0.661	0.181	0.381	3.649	0.001	0.904	1.107	**
Sutra-chanting time (>6 h)	0.090	0.176	0.054	0.513	0.610	0.879	1.138	​
Having leisure activity using voice	0.111	0.194	0.058	0.573	0.569	0.946	1.057	​

​	B	SE	β	t	p	Tolerance	VIF
c							
y-intercept	42.684	25.536	​	1.672	0.100	​	​
Age	0.065	0.085	0.097	0.760	0.450	0.837	1.195
Grip strength (kg)	−0.132	0.148	−0.112	−0.888	0.378	0.848	1.179
Occlusal pressure (N)	−0.001	0.002	−0.104	−0.864	0.391	0.944	1.060
BH (m)	9.695	15.539	0.079	0.624	0.535	0.842	1.187
Smoking habit (brinkman index)	0.004	0.004	0.122	0.983	0.329	0.878	1.139
Being expert priest	3.757	1.895	0.243	1.982	0.052	0.904	1.107
Sutra-chanting time (>6 h)	−2.292	1.838	−0.155	−1.247	0.217	0.879	1.138
Having leisure activity using voice	−0.333	2.034	−0.020	−0.164	0.871	0.946	1.057

B, regression coefficients for non-standardized data; SE, standard error; β, regression coefficients for standardized data; t, t-value; p, p-value; VIF, variance inflation factor; SMI, skeletal muscle mass index; BH, body height.

*p < 0.05; **p < 0.01; ***p < 0.001.

Figure/table caption

There was a mistake in the caption of Table 2 as published. The original content was divided into three separate tables, but during the editing process, they were merged into a single table, which has made the meaning more difficult to understand.

The corrected caption of Table 2 appears below.

“Table 2 Results of the analysis. Effect of expert group in 3 models of multiple regression analyses which includes PEF (Table 2a), FVC (Table 2b), and ⊿HD (Table 2c) as the dependent variables.”

The original article has been updated.

